# Determinants of overall knowledge and health behaviours in relation to hepatitis B and C among ever-married women in Pakistan: evidence based on Demographic and Health Survey 2017–18

**DOI:** 10.1186/s12889-021-12406-z

**Published:** 2021-12-30

**Authors:** Sidra Maqsood, Sarosh Iqbal, Rubeena Zakar, Muhammad Zakria Zakar, Florian Fischer

**Affiliations:** 1 Department of Sociology, Government College University, Lahore, Pakistan; 2 Institute of Social & Cultural Studies, University of the Punjab, Lahore, Pakistan; 3 University of Okara, Okara, Pakistan; 4 Institute of Public Health, Charité – Universitätsmedizin Berlin, Berlin, Germany; 5 Institute of Gerontological Health Services and Nursing Research, Ravensburg-Weingarten University of Applied Sciences, Weingarten, Germany

**Keywords:** HBV, HCV, Knowledge, Attitude, Practice, Social determinants

## Abstract

**Background:**

In 2019, around 5 million and 10 million people were affected by hepatitis B virus (HBV) and hepatitis C virus (HCV) respectively in Pakistan. On World Hepatitis Day 2019, Pakistan’s Government announced the Prime Minister’s Plan to eliminate HBV and HCV from the country by 2030. In order to achieve this goal, adequate knowledge about HBV and HCV regarding mode of transmission, symptoms of the disease, and awareness about available treatments and vaccines is imperative. The present study aims to investigate the determinants related to overall knowledge about and behaviour in relation to HBV and HCV amongst married women in Pakistan.

**Methods:**

Secondary data analysis was carried out using the Pakistan Demographic and Health Survey (PDHS) 2017–18. A series of questions regarding women’s knowledge about how to avoid HBV and HCV and their health behaviour in relation to HBV and HCV were posed to 12,364 ever-married women of reproductive age (15–49 years). Bivariate and multivariable logistic and linear regression was applied to examine the effects of sociodemographic characteristics and covariates on women’s overall knowledge and health behaviour regarding HBV and HCV.

**Results:**

The findings highlight that the majority of women (88.3%) have heard of HBV and HCV. Nonetheless, only 34.8% had comprehensive knowledge about how to avoid HBV and HCV. Few women (11.3%) had been tested for HBV or HCV during the year preceding the survey. Furthermore, the results indicate that women living in urban areas, being older, and having more than 10 years of schooling, reported better knowledge and health behaviours regarding HBV and HCV.

**Conclusion:**

This study provides evidence that women’s sociodemographic characteristics create differences in their overall knowledge about and attitudes towards HBV and HCV. This research emphasized that there is a need to create awareness about the causes and prevention of HBV and HCV in order to achieve the goal of eliminating these diseases in Pakistan by 2030.

## Background

Hepatitis B and C infections cause a range of ailments, ranging from asymptomatic to life-threatening disease [1]. According to estimates by the World Health Organization (WHO), globally 325 million people are affected by hepatitis B virus (HBV) and hepatitis C virus (HCV), resulting in 1.4 million deaths across the world per year. The WHO’s data shows that around 5 million and 10 million people were affected by HBV and HCV respectively in Pakistan during 2019 [2]. A lack of preventive measures, testing services, or treatment resources, the use of unsterilized invasive medical instruments and transfusions of infected blood are the leading causes of high HBV and HCV rates [2, 3]. Furthermore, the data suggests that the primary cause of hepatocellular carcinoma is either hepatitis C or hepatitis B in Pakistan [4]. In October 2017, following the WHO’s Global Health Sector Strategy (GHSS), Pakistan’s Government launched the first National Hepatitis Strategic Framework (2017–2021), aiming to reduce the incidence and chronic cases of hepatitis B and C by 10 and 30%, respectively [2, 5]. In addition to this, on World Hepatitis Day 2019, Pakistan’s Government also announced the Prime Minister’s Plan to eliminate HBV and HCV from the country by 2030. Through this plan, the federal government aims to facilitate provincial governments in providing leadership and coordination to scale up preventive, testing and treatment services for hepatitis [2].

Despite the governmental efforts and commitment, HBV and HCV are still serious public health challenges in Pakistan. Adequate knowledge about HBV and HCV, regarding mode of transmission, symptoms of disease and awareness about available treatments and vaccines is very important for encouraging a favourable attitude towards seeking treatment and avoiding the spread of infections [6, 7]. A previous study conducted in Pakistan indicated that married women had poor and incorrect knowledge about the causes, prevention and treatment of HBV [8]. It is evident through research findings that pregnant women are more at risk, if infected, of transmitting viral hepatitis B and C via sexual, vertical (mother to child during childbirth) and horizontal routes (e.g., blood transfusions and contaminated injections). Therefore, married women’s knowledge about HBV and HCV is crucial for controlling the disease [9, 10].

In light of the above context, there is a dire need for research to understand the determinants of overall knowledge about HBV and HCV among married women. Women who have adequate knowledge about the causes, prevention and treatment of HBV and HCV can be expected to take precautionary measures to avoid the spread of disease and to access treatment services. Previous studies suggest that, in Pakistan, hepatitis B patients lack information about attitudes and practices regarding the prevention of disease [6], and that women have less knowledge about HBV and HCV than men [11]. However, the findings of a study conducted among medical students in Pakistan revealed that female students have more diagnostic knowledge about HBV and HCV than male students [12]. The findings of another study with male respondents revealed that respondents have low levels of knowledge about HBV and HCV, which leads to unfavourable attitudes towards the practising of preventive measures [13].

The studies referred to above with reference to Pakistan were carried out with small sample sizes and lacked evidence of married women’s knowledge and behaviour regarding HBV and HCV. Hence, the present study aims to bridge this research gap, based on a larger sample that is representative of the population of Pakistan. Its prime objective is to investigate various determinants affecting overall knowledge and behaviour relating to HBV and HCV amongst married women in Pakistan.

### Theoretical framework

The theoretical foundation of this research draws upon knowledge, attitude, practice (KAP) theory, which combines aspects of knowledge, attitudes and practice [14], the WHO’s framework of social determinants of health [15] and Diffusion of Innovation (DOI) theory [16].

The assumptions of KAP theory indicate that modifications occur in the health behaviour of individuals after seeking health education and knowledge. It provides a rational model linking health education and behavioural changes [17]. The core postulate of KAP theory emphasizes three successive stages through which an individual’s behaviour passes in order to adapt to changes. These stages are: acquisition of related knowledge, attitude modification and formation of behaviour. Furthermore, KAP theory postulates that acquiring sufficient knowledge regarding symptoms, causes and ways to prevent disease inculcates positive attitudes among individuals about following preventive methods and seeking medical care. Several previous studies have reported that individuals’ KAP level was significantly linked to addressing false perceptions, management of illness, seeking healthcare services and embracing disease-preventive behaviour [18–22].

Furthermore, the social determinants of the health framework emphasize the role of individuals’ sociodemographic factors in determining their health. It indicates that individuals’ health status may vary depending upon their sociodemographic characteristics [23]. The findings of past studies indicate that low socioeconomic strata are linked with low levels of treatment compliance, the inaccessibility of healthcare facilities and insufficient ability to afford medical treatment [24–27]. The literature also reveals that gender, age, level of education and income affect an individual’s knowledge and behaviour in terms of seeking good health [28, 29].

Moreover, the DOI theory postulates that the process of diffusion in society occurs when people learn about innovations. With reference to healthcare, the innovation could be a newly tested approach to improve health system. Furthermore, it argues that the process of diffusion consists of five channels of communication to introduce and advocate an innovation. These channels are knowledge, persuasion, decision, implementation, and confirmation. According to DOI theory, personal characteristics of individuals greatly influence the adoption of innovation. Findings of a previous study highlight that patients with low levels of adoption of medical innovation had a to low socio-economic status [30]. Similarly, literature argues that individuals’ personality traits and socio-economic status are important elements to consider while introducing any innovation [31]. Further, literature supports the application of DOI theory to understand peoples’ perceptions, particularly about disease knowledge, new treatment, awareness raising strategies and effective disease prevention programs. In order to make awareness campaigns more effective, DOI theory encourages the use of advanced technology and mass-media to engage a wider audience [32].

Based on the postulates of KAP theory, the social determinants of health framework and DOI theory, this research explored the social factors linked to the knowledge and behaviour of ever-married women in Pakistan regarding HBV and HCV.

## Methods

### Study design

This research conducted secondary data analysis, using the fourth and latest Pakistan Demographic and Health Survey (PDHS) 2017–18 [33]. Currently, PDHS 2017–18 provides the largest national representative estimates of demographic and health indicators in Pakistan. Furthermore, it provides the largest dataset of variables related to HBV and HCV knowledge among the general population. PDHS 2017–18 is a cross-sectional survey, adopting a stratified two-stage sample design. The stratification was based on urban and rural areas, and a total of 16 strata were obtained, consisting of eight strata each for urban and rural areas. During the first stage, 580 clusters consisting of enumeration blocks were selected, while in the second stage, 16,240 households (28 households per cluster) were selected, using a probability systematic selection process [33].

PDHS 2017–18 collected data on different types of questionnaires. In the present study, data obtained through the women’s questionnaire was used. The women’s questionnaire was administered to 12,364 ever-married women of reproductive age (15–49 years). This women’s questionnaire included questions regarding women’s knowledge and behaviour regarding hepatitis B and C [33].

### Outcome variables

In this study, women’s awareness, knowledge/health beliefs about HBV or HCV, and health behaviour are taken as outcome variables. Initially, women’ awareness was explored, if they had ever heard of illnesses called HBV or HCV (“Yes”/“No”). Women who replied “yes” were asked further questions to inquire about their knowledge and health behaviour regarding HBV or HCV.

Women’s overall knowledge or health beliefs about HBV or HCV was inferred from the following question: “Is there anything a person can do to avoid getting HBV or HCV?” (“Yes”/“No”/“Don’t know”). Women who replied “yes” were further asked: “What can a person do to avoid getting HBV or HCV?” Possible responses to this question included six options: “practice safe sex”, “safe blood transfer”, “use disposable syringe”, “avoid contaminated food/water”, “avoid contact with infected person”, and “ensure dentists’ instruments properly sterilized”. Respondents who replied correctly were coded as 1, while those who replied incorrectly or “don’t know” were coded as 0. Hence, the score for women’s knowledge about HBV or HCV ranged from 0 to 6. For this particular research, the women’s knowledge about HBV or HCV was used as both continuous (0–6 score) and binary variable, where the score was dichotomized into no knowledge (score 0) vs. some knowledge (score 1–6). The reason of selecting dichotomous or binary variables is based on running the logistic regression, which is widely used in public health research. However, considering the significance of original 6-points women’s knowledge scale, a need was also felt to apply it as continuous variable to avoid reduction in precision and statistical power and misinterpretation of the effects of degrees of knowledge. Furthermore, this research finds it more beneficial and powerful to see the nuanced effects of dependent variables as both continuous and dichotomous variables.

Health behaviour was determined through the following question: 1) “Have you ever been tested for HBV or HCV?” (“Yes”/“No”). 2). Those respondents, who reported “yes” were further asked about their recent test: “How many months ago was your most recent test for HBV or HCV?” (“Within the last year”/“More than one years ago”).

### Independent variables

In this study, women’s healthcare decision-making autonomy, exposure to mass media to access information, health insurance coverage and the accessibility of distant healthcare facilities were taken as independent variables, which may influence women’s knowledge and behaviour about HBV or HCV.

Women’s healthcare autonomy measures their overall contribution towards decisions about healthcare (“Who usually decides about your healthcare?”). Possible responses were: “respondent alone”, “husband/partner alone”, “respondent and husband/partner jointly”, “respondent and other person”, and “someone else or others”. Since there was no response against ‘respondent and other person’, hence, for this study, responses were categorized into four groups: a) woman/respondent alone, b) husband alone, c) woman and husband jointly, and d) someone else. A covariate of exposure to mass media to access information measures the women’s frequency of reading a newspaper, watching TV or listening to the radio, and was grouped into two categories (“Yes”/“No”). Furthermore, women were asked about health insurance coverage (“Yes”/“No”) and the accessibility of distant healthcare facilities for seeking medical services (“big problem”/“not a big problem”).

A set of sociodemographic variables was included: geographical classification (“urban”/“rural”), region/province (Punjab; Sindh; Khyber Pakhtunkhwa; Baluchistan; Gilgit Baltistan; Islamabad), age of respondents (in years), educational status of respondents and husbands, employment/occupation of respondents and husbands, household wealth quintile. Women’s age was grouped into three categories (“15–24 years”/“25–34 years”/“35 years and above”). The educational status of women and their spouses/husbands was grouped into four categories (“no formal schooling”/“up to 5 years of schooling”/“6–10 years of schooling”/“more than 10 years of schooling”). The employment/occupation of respondents and their spouses/husbands was grouped into four categories (“not working or unemployed”/“professional, clerical, sales & services”/ “agriculture”/“manual or household worker”).

Additionally, women’s knowledge about HBV or HCV, respondents’ age and education, and their spouses/husbands’ years of education attainment were also taken as continuous variables.

### Statistical analysis

SPSS version 21 was used for the data analysis. We applied sampling weights for all analyses. Descriptive statistics were employed for sociodemographic characteristics. Measures of women’s awareness, overall knowledge and health behaviour regarding hepatitis B or C, women’s healthcare decision-making autonomy, exposure to mass media, access to a distant health facility and health insurance coverage were compiled in the form of frequencies and percentages. Further, means and standard deviations (SD) were also presented for respondents’ knowledge, age, and education. The chi-square test was applied to examine the association and determine a *p*-value for cross-tabulation. An association was considered significant when the p-value was < 0.05.

Both logistic and linear regression modelling was performed for this particular research. Simple bivariate and multivariate linear regression was performed to examine the effects of women’s knowledge about HBV and HCV related to key characteristics. Findings of linear regression are presented as β coefficients with standard error (SE), R^2^ and 95% Confidence Interval (CI). Furthermore, bivariate and multivariable logistic regression models were also applied to investigate the effect of sociodemographic characteristics and further independent variables on women’s behaviour related to HBV and HCV. The results of logistic regression are presented as odds ratios (OR) and adjusted odds ratios (AOR) with 95% CI.

In multivariate regression modelling, accessibility to a distant health facility was adjusted and fixed as confounding variable. Further, multicollinearity between variables was also examined using a variance inflation factor (VIF). During logistic regression modelling, no multicollinearity was observed. However, higher values of VIF > 10 [34] were found during the linear regression modelling for the province of Punjab, Sindh and Khyber Pakhtunkhwa, which were eliminated from the multivariate linear model.

## Results

### Sociodemographic characteristics and covariates

Table 1 describes the sociodemographic characteristics and covariates of the sample of 12,364 ever-married women. The majority resided in rural areas (63.2%) and belonged to the age group 25–34 years (40.1%). With reference to educational status, the majority of respondents (49.2%) had no formal schooling, while 21.2% had completed 6–10 years of schooling, and only 13.1% had attained more than 10 years of schooling. The majority of respondents (80.1%) were unemployed. Regarding husbands’ education, a higher proportion of respondents informed that their husbands attended schools (95.4%) and attained education between 6 to 10 years of schooling (35.3%). Further, the majority of respondents highlighted that their husbands had worked in last 12 months (95.5%) and served as manual or household workers (45.1%) or were associated with professional, clerical, sales and services (33.7%). Most of the respondents (66%) had access to mass media. With reference to healthcare decision-making and autonomy, only 9.6% of the respondents had autonomy, whereas 37.2% reported that their husbands make decisions. However, 41% of the respondents reported that they jointly (both respondent and husband) make decisions regarding healthcare services. Furthermore, a significant percentage (42.0%) of respondents reported that the accessibility of distant healthcare facilities for seeking medical services was a problem, and almost all respondents (98.6%) had no health insurance coverage.

**Table 1:** Sample description (*n* = 12,364)

Characteristics	n	%
**Sociodemographic characteristics**		
**Regions / Provinces**		
Punjab	6630	53.6
Sindh	2850	23.1
Baluchistan	642	5.2
Khyber Pakhtunkhwa	1901	15.4
Islamabad Capital Territory	107	0.8
FATA	234	1.9
**Geographical classification**		
Urban	4550	36.8
Rural	7814	63.2
**Respondent’s age**		
M (SD)	32.27 (8.35)	
15–24 years	2489	20.1
25–34 years	4961	40.1
35 years and above	4914	39.8
**Respondent’s educational status**		
M (SD)	4.36 (5.05)	
No formal schooling	6080	49.2
Up to 5 years of schooling	2037	16.5
6–10 years of schooling	2623	21.2
More than 10 years of schooling	1624	13.1
**Respondent’s occupation**		
Not working / unemployed	9894	80.1
Professional, clerical, sales & services	648	5.2
Agriculture	778	6.3
Manual or Household worker	1034	8.4
**Husband ever attended school**		
Yes	11,795	95.4
No	537	4.3
Don’t know	32	0.3
**Husband’s educational status**^**a**^		
M (SD)	6.49 (5.11)	
No formal schooling	3480	29.5
Up to 5 years of schooling	1840	15.6
6–10 years of schooling	4165	35.3
More than 10 years of schooling	2310	19.6
**Husband has worked in last 12 months**		
Yes, worked	11,806	95.5
No, didn’t work	535	4.3
Don’t know	23	0.2
**Husband’s occupation**^**a**^		
Not working / unemployed	489	4.1
Professional, clerical, sales & services	3980	33.7
Agriculture	2013	17.1
Manual or household worker	5324	45.1
**Wealth quintile**		
Richest	2579	20.9
Richer	2594	21
Middle	2504	20.2
Poorer	2430	19.6
Poorest	2257	18.3
**Covariates**		
**Exposure to mass media / Access to information**		
Yes	8153	66.0
No	4200	34.0
**Healthcare decision-making / Autonomy**^**a**^		
Respondent alone	1132	9.6
Respondent and husband/partner	4847	41
Husband/partner alone	4404	37.2
Someone else	1446	12.2
**Accessibility to distant health facility for seeking medical services**		
Big problem	5191	42.0
Not a big problem	7164	58.0
**Health insurance coverage**		
Yes	177	1.4
No	12,187	98.6

^**a**^ Data does not add up to 100%, due to missing data (< 5%), which arouse from not applicable data and the response category “Don’t know”

### Awareness, overall knowledge and health behaviour

**Fig. 1:**
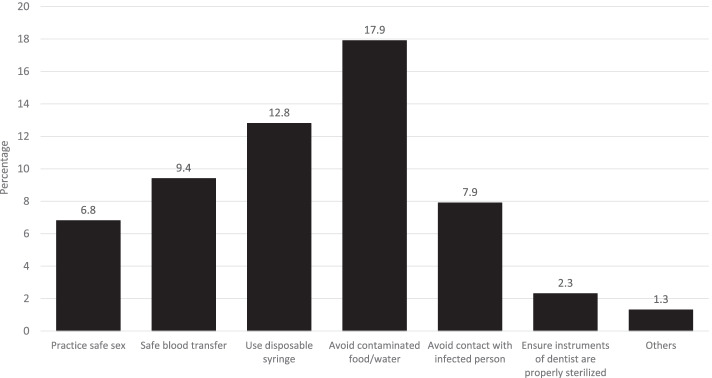
Awareness about various ways to avoid hepatitis B or C

The majority of respondents (88.3%) were aware of HBV and HCV. With reference to women’s knowledge of HBV and HCV, it was found that more than half of respondents (57.4%) were aware of various ways to avoid HBV and HCV. However, only some of the respondents (34.8%) were found to have comprehensive knowledge about how to avoid HBV and HCV (Table 2). Upon probing, the results showed that less than one-fifth of women reported contaminated water and food (17.9%) or use of an infected syringe (12.8%) as the main risk factors for HBV and HCV. Furthermore, some of the women were also found to be aware of safe sex practices (6.8%), safe blood transfusion (9.4%), and refraining from contact with an infected person (7.9%) to avoid HBV and HCV (Fig. 1).

**Table 2:** Awareness, knowledge and health behaviour regarding hepatitis B or C (*n* = 12,364)

Characteristics	f	%
**Women has ever heard of hepatitis B or C (n = 12,364)**		
Yes	10,919	88.3
No	1445	11.7
**Women’s knowledge of hepatitis B or C**		
**Women’s awareness about various ways to avoid hepatitis B or C**^**a**^ **(*n*** **= 10,919)**		
Yes	4654	57.4
No	6264	42.6
**Women’s comprehensive knowledge to avoid hepatitis B or C**^**a**^ **(*n*** **= 10,919)**		
M (SD)	0.58 (0.95)	
Yes	3804	34.8
No	7114	65.2
**Women’s health behaviour towards hepatitis B or C**^**a**^		
**Ever been tested for hepatitis B or C**^**a**^**(*n*** **= 10,919)**		
Yes	3174	25.7
No	7745	62.6
**Months ago tested for hepatitis B or C**^**b**^ **(*n*** **= 3174)**		
Within last year (last 12 months)	1437	45.3
More than 1 year ago	1735	54.7

^a^ Those women, who had ever heard of HBV or HCV, i.e. 88% of the sample were asked further questions about their knowledge and health behaviour including testing for HBV or HCV

^**b**^ Those women who had ever been tested for HBV or HCV (i.e. 25.7% of the sample) were asked further probing question about how many months passed since the test for HBV or HCV

With reference to women’s health behaviour towards HBV and HCV, some of the respondents (25.7%) had been tested for HBV and HCV at some point (Table 2).

### Association of women’s overall knowledge and health behaviour regarding HBV and HCV with respondents’ characteristics

Respondents’ overall knowledge about HBV and HCV was found to be high among respondents from Islamabad Capital Territory (56.1%) and Sindh (41.0%), those living in urban areas (46.0%), in the age group of 35 years and above (38.4%), with more than 10 years of schooling (62.5%), working in professional, clerical, sales & services jobs (52.2%), and belonging to the richest wealth quintile (55.3%). Respondents having mass media exposure (39.9%) indicated better knowledge about HBV and HCV than their counterparts. With reference to healthcare decision-making and autonomy, the categories of respondents alone (39.5%) and jointly, both respondent and husband (39.8%) reported better knowledge about HBV and HCV. Furthermore, the overall knowledge about HBV and HCV was found to be high amongst those respondents who had access to distant healthcare facilities for medical services (38.8%) and health insurance coverage (41.3%). Similar trends were also found in the results for respondents’ health behaviour relating to HBV and HCV.

A statistically significant association (*p* < 0.05) of both variables, i.e. overall knowledge and health behaviour relating to HBV and HCV, was observed with respondents’ age, the educational status of respondents and their husbands, the occupation of respondents and their husbands, wealth quintile, exposure to mass media, autonomy to make decisions about healthcare, accessibility of distant healthcare facilities and health insurance coverage (Table 3).

**Table 3:** Association of women’s knowledge and health behaviour on hepatitis B or C with sociodemographic characteristics and covariates (*n* = 10,919)

Characteristics	Knowledge about hepatitis B or C			Health behavior about hepatitis B or C		
	Yes	No	***p***-value*	Yes	No	***p***-value*
**Regions / Provinces**						
Punjab	34.2	65.8	**< 0.01**	29.8	70.2	**< 0.01**
Sindh	41	59		34.8	65.2	
Baluchistan	28.3	71.7		18.2	81.8	
Khyber Pakhtunkhwa	31	69		21.9	78.1	
Islamabad Capital Territory	56.1	43.9		38.8	61.2	
FATA	17.6	82.4		19	81	
**Geographical classification**						
Urban	46	54	**< 0.01**	38.1	61.9	**< 0.01**
Rural	28	72		23.5	76.5	
**Respondent’s age**						
15–24 years	25.2	74.8	**< 0.01**	18.2	81.8	**< 0.01**
25–34 years	35.8	64.2		31.6	68.4	
35 years and above	38.4	61.6		31.6	68.4	
**Respondent’s educational status**						
No formal schooling	23.9	76.1	**< 0.01**	22	78	**< 0.01**
Up to 5 years of schooling	33.4	66.6		26.2	73.8	
6–10 years of schooling	40.9	59.1		33.1	66.9	
More than 10 years of schooling	62.5	37.5		48.8	51.2	
**Respondent’s occupation**						
Not working / unemployed	35.1	64.9	**< 0.01**	29.1	70.9	**< 0.01**
Professional, clerical, sales & services	52.2	47.8		41.8	58.2	
Agriculture	16.9	83.1		17.8	82.2	
Manual or household worker	33.6	66.4		28.7	71.3	
**Husband’s educational status**						
No formal schooling	23.8	76.2	**< 0.01**	19.9	80.1	**< 0.01**
Up to 5 years of schooling	30.2	69.8		27.6	72.4	
6–10 years of schooling	35.3	64.7		29.3	70.7	
More than 10 years of schooling	53	47		42.2	57.8	
**Husband’s occupation**						
Not working / unemployed	29.4	70.6	**< 0.01**	23.8	76.2	**< 0.01**
Professional, clerical, sales & services	44.3	55.7		37.3	62.7	
Agriculture	24.2	75.8		23	77	
Manual or household worker	32.1	67.9		25.6	74.4	
**Wealth quintile**						
Richest	55.3	44.7	**< 0.01**	43.9	56.1	**< 0.01**
Richer	38.1	61.9		33.6	66.4	
Middle	32.2	67.8		26.1	73.9	
Poorer	23	77		18.9	81.1	
Poorest	19.9	80.1		18.6	81.4	
**Exposure to mass media / Access to information**						
Yes	39.9	60.1	**< 0.01**	32.9	67.1	**< 0.01**
No	24.1	75.9		21.0	79.0	
**Healthcare decision-making / Autonomy**						
Respondent alone	39.5	60.5	**< 0.01**	33.3	66.7	**< 0.01**
Respondent and husband/partner	39.8	60.2		31.7	68.3	
Husband/partner alone	31.3	68.7		27.5	72.5	
Someone else	25.4	74.6		21.3	78.7	
**Accessibility to distant health facility for seeking medical services**						
Big problem	29.1	70.9	**< 0.01**	23.8	76.2	**< 0.01**
Not a big problem	38.8	61.2		32.8	67.2	
**Health insurance coverage**						
Yes	41.3	58.7	0.07	42.4	57.6	**< 0.01**
No	34.7	65.3		28.9	71.1	

* Chi-square test was applied to determine *p*-value

### Bivariate and multivariable regression

The results of simple bivariate and multivariable linear regression models of women’s knowledge on BV or HCV (Table 4) and multivariable logistic regression models of women’s health behaviour (Table 5) related to sociodemographic characteristics and covariates are presented below.

**Table 4:** Simple bivariate and multivariable linear regression models of women’s knowledge on hepatitis B or C (*n* = 10,919)

Characteristics	Women’s knowledge about hepatitis B or C					
	Bivariate			Multivariate		
	***R***^**2**^	β (SE)	***p***-value	β (SE)	CI (95%)	***p***-value
**Regions / Provinces**						
FATA	1			1		
Punjab^**a**^				–	–	–
Sindh^**a**^	0.01	0.26 (0.02)	**< 0.01**	–	–	**–**
Baluchistan	0.001	0.18 (0.05)	**< 0.01**	0.31 (0.09)	0.14–0.48	**< 0.01**
Khyber Pakhtunkhwa^**a**^	0.001	−0.08 (0.03)	**< 0.01**	–	–	**–**
Islamabad Capital Territory	0.001	0.38 (0.09)	**< 0.01**	0.07 (0.12)	− 0.16-0.29	0.57
**Geographical classification**						
Rural	1			1		
Urban	0.03	0.35 (0.02)	**< 0.01**	0.11 (0.03)	0.06–0.167	**< 0.01**
**Respondent’s age**	0.01	0.01 (0.001)	**< 0.01**	0.01 (0.002)	0.01–0.02	**< 0.01**
**Respondent’s educational status**	0.07	0.05 (0.002)	**< 0.01**	0.04 (0.003)	0.03–0.04	**< 0.01**
**Husband’s educational status**	0.04	0.04 (0.002)	**< 0.01**	0.01 (0.003)	0.01–0.02	**< 0.01**
**Respondent’s occupation**						
Not working / unemployed	1			1		
Professional, clerical, sales & services	0.01	0.38 (0.04)	**< 0.01**	0.18 (0.05)	0.08–0.28	**< 0.01**
Agriculture	0.01	−0.39 (0.04)	**< 0.01**	−0.08 (0.07)	− 0.21- 0.05	0.23
Manual or household worker	0.00	−0.003 (0.03)	0.91	0.05 (0.04)	−0.04-0.14	0.29
**Husband’s occupation**						
Not working / unemployed	1			1		
Professional, clerical, sales & services	0.02	0.28 (0.02)	**< 0.01**	0.00 (0.06)	−0.12-0.13	0.99
Agriculture	0.01	−0.21 (0.03)	**< 0.01**	−0.14 (0.07)	− 0.28-(− 0.003)	**0.04**
Manual or household worker	0.003	−0.11 (0.02)	**< 0.01**	− 0.05 (0.06)	− 0.18-0.06	0.34
**Wealth quintile**						
Poorest	1			1		
Poorer	0.01	−0.26 (0.02)	**< 0.01**	−0.12 (0.05)	− 0.21-(− 0.02)	**0.02**
Middle	0.001	−0.09 (0.02)	**< 0.01**	− 0.13 (0.05)	− 0.23-(− 0.03)	**0.01**
Richer	0.00	0.04 (0.02)	**0.05**	−0.20 (0.05)	−0.30-(− 0.10)	**< 0.01**
Richest	0.05	0.51 (0.02)	**< 0.01**	0.01 (0.06)	−0.12-0.11	0.89
**Healthcare decision-making / Autonomy**						
Someone else	1			1		
Respondent alone	0.001	0.08 (0.03)	**0.01**	0.14 (0.05)	0.04–0.25	**0.01**
Respondent and husband/partner	0.01	0.15 (0.02)	**< 0.01**	0.16 (0.04)	0.08–0.24	**< 0.01**
Husband/partner alone	0.002	−0.08 (0.02)	**< 0.01**	0.10 (0.04)	0.01–0.19	**0.02**
**Exposure to mass media**						
No	1			1		
Yes	0.02	0.28 (0.02)	**< 0.01**	0.04 (0.03)	−0.02-0.10	0.19
**Health insurance coverage**						
No	1			1		
Yes	0.001	0.18 (0.07)	**0.01**	0.15 (0.09)	−0.04-0.34	0.13
**Accessibility to distant health facility for seeking medical services**^**b**^						
Big problem	1			–	–	–
Not a big problem	0.01	0.19 (0.02)	**< 0.01**	–	–	–
**Model for good fit (R**^**2**^**)**				**0.119**		

^a^ VIF > 10 was observed, indicating multicollinearity, so eliminated from multivariate modeling

^b^ Multivariate modeling was adjusted for the confounding variable of accessibility to a distant health facility for seeking medical services

**Table 5:** Bivariate and multivariable logistic regression models of women’s health behaviour on hepatitis B or C (*n* = 10,919)

Characteristics	Health behaviour about hepatitis B or C					
	Bivariate			Multivariate		
	OR	95% CI	***p***-value	AOR	95% CI	***p***-value
**Regions / Provinces**						
FATA	1			1		
Punjab	1.78	1.27–2.50	**< 0.01**	0.66	0.28–1.58	0.35
Sindh	2.24	1.59–3.17	**< 0.01**	0.94	0.39–2.24	0.89
Baluchistan	0.94	0.62–1.42	0.77	0.37	0.14–0.98	**0.04**
Khyber Pakhtunkhwa	1.18	0.83–1.68	0.36	0.49	0.21–1.18	0.11
Islamabad Capital Territory	2.64	1.56–4.48	**< 0.01**	0.73	0.27–1.99	0.54
**Geographical classification**						
Rural	1			1		
Urban	2.01	1.84–2.18	**< 0.01**	1.12	0.97–1.28	0.12
**Respondent’s age**	1.02	1.02–1.03	**< 0.01**	1.02	1.01–1.03	**< 0.01**
**Respondent’s educational status**	1.09	1.08–1.09	**< 0.01**	1.04	1.03–1.06	**< 0.01**
**Husband’s educational status**	1.08	1.07–1.09	**< 0.01**	1.03	1.01–1.04	**< 0.01**
**Respondent’s occupation**						
Not working / unemployed	1			1		
Professional, clerical, sales & services	1.75	1.48–2.06	**< 0.01**	1.23	0.98–1.53	0.07
Agriculture	0.52	0.43–0.64	**< 0.01**	0.86	0.61–1.22	0.41
Manual or household worker	0.98	0.84–1.14	0.81	1.08	0.87–1.33	0.50
**Husband’s occupation**						
Not working / unemployed	1			1		
Professional, clerical, sales & services	1.90	1.51–2.39	**< 0.01**	0.96	0.71–1.31	0.81
Agriculture	0.95	0.74–1.22	0.71	0.73	0.52–1.03	0.08
Manual or household worker	1.09	0.87–1.38	0.43	0.85	0.63–1.15	0.30
**Wealth quintile**						
Poorest	1			1		
Poorer	1.02	0.87–1.19	0.81	1.12	0.86–1.45	0.41
Middle	1.54	1.33–1.80	**< 0.01**	1.11	0.85–1.44	0.43
Richer	2.21	1.91–2.55	**< 0.01**	1.36	1.04–1.78	**0.03**
Richest	3.42	2.96–3.94	**< 0.01**	1.53	1.14–2.07	**< 0.01**
**Healthcare decision-making / Autonomy**						
Someone else	1			1		
Respondent alone	1.84	1.53–2.22	**< 0.01**	1.28	0.99–1.66	0.06
Respondent and husband/partner	1.71	1.47–1.99	**< 0.01**	1.26	1.02–1.56	**0.04**
Husband/partner alone	1.40	1.20–1.63	**< 0.01**	1.27	1.02–1.58	**0.03**
**Exposure to mass media**						
No	1					
Yes	1.84	1.67–2.02	**< 0.01**	0.95	0.81–1.11	0.54
**Health insurance coverage**						
No	1			1		
Yes	1.82	1.34–2.47	**< 0.01**	1.19	0.77–1.86	0.42
**Accessibility to distant health facility for seeking medical services**^**a**^						
Big problem	1			–		
Not a big problem	1.57	1.44–1.71	**< 0.01**			

^a^ Multivariate modeling was adjusted for the confounding variable of accessibility to a distant health facility for seeking medical services

Table 4 shows that in bivariate analysis, a statistically significant association (*p* < 0.05) was observed with region, place of residence, age, respondents and their husbands’ educational status and occupation, wealth index, healthcare decision making autonomy, exposure to mass media, health insurance coverage and accessibility to a distant health facility for seeking medical services.

In multivariate linear regression, Baluchistan province was found to be significantly associated with women’s knowledge related to HBV or HCV. It reveals that the knowledge increased to 0.31 (95% CI: 0.14–0.48) amongst women. However, no association was seen with women living in Islamabad Capital Territory. It is pertinent to mention that due to high multicollinearity, the province of Punjab, Sindh and Khyber Pakhtunkhwa were excluded from multivariate analysis. Similarly, women belonging to urban areas had 0.11 (95% CI: 0.06–0.17) increased knowledge for HBV or HCV (Table 4). Furthermore, results revealed that women’s knowledge had more likelihood to increase to 0.01 (95% CI: 0.01–0.02) with age, 0.04 (95% CI: 0.03–0.04) with respondents’ educational status and 0.01 (95% CI: 0.01–0.02) with husbands’ educational status. With reference respondents’ occupation, knowledge increased to 0.18 (95% CI: 0.08–0.28) among women belonging to the category of professional/clerical/sales & services. Knowledge increased to 0.16 (95% CI: 0.08–0.24) among those respondents who take healthcare decisions along with their husbands. Furthermore, it was found that women who had exposure to media are more likely to have a higher knowledge (0.04; 95% CI: − 0.02–0.10) and women with health insurance coverage had increased knowledge to 0.15 (95% CI: − 0.04–0.34).

The logistic regression results presented in Table 5, related to factors associated with health behaviour, are generally comparable to the results for overall knowledge, showing significant association with respondents living in Baluchistan, age, education and their husbands’ educational status. However, respondents’ wealth quintile showed a significant difference for richer (AOR = 1.36, 95% CI: 1.04–1.78) and richest (AOR = 1.53, 95% CI: 1.14–2.07) compared to the categories of poor, poorer and middle. With reference to healthcare decision-making/autonomy, multivariate analysis indicates that women who can take decisions jointly with their husbands (AOR = 1.26, 95% CI: 1.02–1.56), and whom husbands take decisions alone (AOR = 1.27, 95% CI: 1.02–1.58) are more likely to have positive health behaviours related to HBV and HCV.

## Discussion

This study set out to explore the relationship of sociodemographic characteristics and other variables with women’s overall knowledge and health behaviour related to HBV and HCV. The findings highlight that the majority of women (88.3%) had heard about HBV and HCV. Nonetheless, only 34.8% had comprehensive knowledge about how to avoid HBV and HCV. With regard to women’s awareness of various ways to avoid HBV and HCV, our study revealed that the majority of women knew that contaminated water and food, as well as use of infected syringes, are risk factors for HBV and HCV. Furthermore, some of the women were also aware of safe sex practices, safe blood transfusions and avoiding contact with infected persons to avoid HBV and HCV. Since the reported percentage of women’s awareness is low, these findings highlight the importance of increasing awareness campaigns among the general public and to encourage people to go for testing in order to implement Pakistan’s Government Plan to eliminate HBV and HCV by 2030 [2]. These findings are in line with previous research conducted in Ethiopia, which reported that the majority of women had never been screened for HBV [35]. The findings from a study in Ghana revealed that slightly fewer than half of the women surveyed reported knowledge about HBV [36]. Another small-scale study conducted in Pakistan reported that the majority of both men and women (75.4%) have poor knowledge regarding HBV and have never gone through HBV testing (96.9%) [6].

With reference to the association between women’s overall knowledge and health behaviour regarding HBV and HCV and sociodemographic characteristics, the present study indicated that women living in urban areas, with higher age, better education, higher income and employed in the group of professional, clerical, sales & services reported better overall knowledge and health behaviour regarding HBV and HCV. These results are comparable with other studies carried out in Pakistan [11, 37], India [38], Uganda [39] and Poland [40]. Furthermore, these findings are also in line with the framework of social determinants of health, which emphasizes that an individual’s socioeconomic position is (positively or negatively) associated with their knowledge and health behaviour [23]. Furthermore, the findings are consistent with previous studies based on DOI theory which emphasized that individuals belonging to urban regions and having better education have a higher capability to adopt innovations in healthcare [30, 31].

In addition to sociodemographic factors, the findings of this study revealed that exposure to mass media and autonomy to make decisions about personal healthcare were positively associated with good health behaviour related to HBV and HCV. These findings are consistent with the results of previous studies conducted in Poland [40] and Pakistan [41], which highlighted women’s autonomy and the positive effect of the media on knowledge and awareness about disease prevention. Here, it is also important to note that the media has two important responsibilities in order to play its part in disease prevention. Firstly, it is responsible for creating awareness regarding the causes of disease, available treatments and measures to prevent the disease. Secondly, it is responsible for countering infodemics by avoiding misinformation regarding any health-related issue [42]. Literature based on DOI theory also encourages the use of advanced technology and mass media to make awareness campaigns more effective [32].

Summing up, the findings highlight the importance of obtaining accurate and comprehensive knowledge regarding the prevention and treatment of HBV and HCV. Regarding knowledge and awareness, it is important that reliable and valid knowledge is imparted to married women to bring about change in their attitudes and behaviour in relation to disease [43, 44]. It is evident from various studies that accurate and reliable knowledge helps to reduce myths and doubts associated with HBV and HCV [45–47]. Similarly, reliable sources may be used to disseminate information and knowledge regarding HBV. This information may help in shaping and moulding the attitudes and behaviour of individuals. Therefore, suitable and effective health education programmes and behaviour-change strategies at a community level should be launched in order to reduce misconceptions and help women to access services.

### Strengths and limitations

This study has its own strengths and limitations. An important strength is the use of nationally representative data from PDHS to investigate the determinants of overall knowledge and health behaviours in relation to HBV and HCV among ever-married women in Pakistan. Furthermore, the findings of the study are supported by a proposed theoretical framework. Despite the strengths of this study, such as the large sample size and nationally representative data, one needs to consider several limitations when interpreting the data. A major limitation is the fact that the analysis is based on secondary data, which does not include all the items relevant to a holistic view on the determinants of knowledge and behaviour related to HBV and HCV. Furthermore, the assessment does not distinguish between HBV and HCV and combines these two diseases. Additionally, it is a cross-sectional design which does not allow for the investigation of causal relationships.

## Conclusion

This study provides evidence that women’s sociodemographic characteristics are associated with knowledge and behaviours relating to HBV and HCV. There is a need to create awareness about the causes and prevention of HBV and HCV among women residing in rural areas and having less education. In order to achieve the goal to eliminate HBV and HCV in Pakistan by 2030, the government needs to launch comprehensive mass media campaigns, particularly in rural areas, to provide adequate information about HBV and HCV regarding mode of transmission, symptoms of disease and awareness about available treatments and vaccines in order to inculcate favourable attitudes towards seeking treatment and to reduce the spread of infections. The medium of interpersonal communication may be utilized to engage community at wider level. It is essential to design and distribute information, education, and communication materials to organize community meetings, events and seminars to enhance HBV and HCV related knowledge and behaviour. Furthermore, user-friendly and community need-based informative messages in local languages may also be disseminated for public awareness and promotion of best practices.

## Data Availability

Data is publicly available from https://dhsprogram.com/.

## Abbreviations

AOR: Adjusted odds ratio
CI: Confidence interval
DOI: Diffusion of Innovation
HBV: Hepatitis B virus
HCV: Hepatitis C virus
GHSS: Global Health Sector Strategy
KAP: Knowledge, attitude, practice
OR: Odds ratio
PDHS: Pakistan Demographic and Health Survey
SPSS: Statistical Package for the Social Sciences
VIF: Variance inflation factor
WHO: World Health Organization
